# The threat of antimicrobial resistance in surgical care: the surgeon’s role and ownership of antimicrobial stewardship

**DOI:** 10.1093/bjs/znad302

**Published:** 2023-09-27

**Authors:** Gabriel Birgand, Puneet Dhar, Alison Holmes

**Affiliations:** National Institute for Health Research Health Protection Research Unit in Healthcare Associated Infections and Antimicrobial Resistance, Imperial College London, London, UK; Regional Center for Infection Prevention and Control, Region of Pays de la Loire, Nantes University Hospital, Nantes, France; Cibles et médicaments des infections et de l'immunité, IICiMed, UR 1155, Nantes Université, Nantes, France; Surgical Gastroenterology, Amrita Hospital, Faridabad, India; National Institute for Health Research Health Protection Research Unit in Healthcare Associated Infections and Antimicrobial Resistance, Imperial College London, London, UK; Faculty of Health Sciences, University of Liverpool, Liverpool, UK

Postoperative infections and antimicrobial resistance (AMR) are inseparable threats. AMR poses a societal-level hazard, with an estimated 1.27 million deaths attributable to bacterial AMR globally in 2019^1^. *Escherichia coli* and *Staphylococcus aureus*, the two leading pathogens for deaths associated with antibacterial resistance, are also the two most prevalent bacterial species isolated from postoperative infections. The miracle impact of antibiotics to reduce infectious morbidity in surgery is rapidly waning.

The global incidence of surgical site infections (SSIs) is estimated by WHO to be 3–50 per cent, depending on the type of surgery, and is particularly high in low- and middle-income countries (LMICs), where SSIs are the most frequently reported healthcare-associated infection (HAI)^2^. The incidence of caesarean section SSI is estimated to 5.6 per cent globally, significantly higher in African regions (11.9 per cent) and steadily increasing since the COVID-19 pandemic^3^. Other postoperative infection that occurs beyond the incision site (for example, pneumonia, urinary tract infection, bacteraemia) represents a substantial burden and safety concern in perioperative care. In African countries, infection occurs in 10.2 per cent of patients following surgery, representing the most common postoperative complication^4^. Reported figures may underestimate the burden because of poor documentation and surveillance.

The capacity of healthcare systems to deliver safe surgery depends on access to effective antibiotics, particularly for prophylaxis in many clean, clean-contaminated and contaminated procedures^2^. Contracting an SSI increases antibiotic use seven-fold^5^. Up to 60 per cent of surgical patients receive postoperative antibiotics while in hospital, and up to 50 per cent are discharged on antibiotics^6^. The overuse and inappropriate use of antibiotics for prophylaxis and therapy remain areas of concern, amplifying the AMR problem. AMR threatens society’s long-term capacity to effectively deliver high-quality perioperative care and healthcare overall. Burn care serves as a compelling illustration of how AMR can impact surgical care with high prevalence of multi-drug–resistant bacteria, high antibiotic and colonization pressures, need for intensive medical and surgical therapy, in a vulnerable and immunocompromised patient population.

Preventing postoperative infections significantly reduces the need for antimicrobials in acute care, and is essential to mitigate the global burden of AMR. Although evidence-based guidelines for SSI prevention are well established, the compliance with best practices including surgical antibiotic prophylaxis (SAP) remains suboptimal. Inappropriate drug choice (for example, unnecessary vancomycin use, patient allergy status) and the timely administration and stopping of SAP (for example, extended SAP beyond the surgical procedure in 80 per cent of rural hospitals in India) increase the risk of SSI and harm and promote AMR^7^. The background prevalence of AMR in the general population also impacts the efficacy of SAP. Colonization with extended spectrum beta-lactamase producing Enterobacterales more than doubles the SSI rates in gastrointestinal surgery for patients given routine SAP^8^. In a multicentre study, 21.6 per cent of SSIs post gastrointestinal surgery were with an organism resistant to the SAP administered, varying from 16.6 per cent in high-income countries (HICs) to 36 per cent in low-income countries (LICs)^9^. Recent guidelines therefore recommend rectal screening for resistant Enterobacterales and targeted SAP before transrectal ultrasound-guided prostate biopsy, colorectal surgery or solid organ transplantation^10^.

SSI prevention also involves the skin preparation, and in some cases the preoperative decolonization with topical antibiotics as an adjunct to SAP. However, the potential collateral damage on the microbiome they may cause is still questioned. Vaccines, passive immunization and advances in antibody-based approaches are attractive potential alternatives or additions to decrease the overall burden of postoperative infection. However, vaccine development for HAIs has been hampered by many factors such as the immune response of at-risk patients, the commensal form of targeted pathogens such as *S. aureus*^11^. For most pathogens involved in SSI, vaccines are not currently available, and the development of effective vaccines for *S. aureus* or Gram-negative bacteria remains challenging. Despite still little clinical evidence, bacteriophages represent a promising option offering a pathogen-specific antibacterial alternative to antibiotics in surgery.

Although more antibiotics are prescribed for surgical than medical patients, insufficient antimicrobial stewardship (AMS) efforts have been made on surgical specialties^6^. Antibiotic prescribing in hospital settings is ubiquitous and doctors of different specialties must be able to diagnose and treat infections. Improved monitoring and feedback of SSI incidence and postoperative antibiotic use to all perioperative care providers should be prioritized and used as an indicator of quality care^12^. Researchers in India and the UK have highlighted the lack of clarity regarding the ownership of the antibiotic prescription, particularly in surgical pathways where multiple healthcare professionals have a role to play^13^. The confusion around medical decision making for treating infections in surgical patients leads to uncoordinated and suboptimal antimicrobial management. The power dynamics that exist in surgical teams in relation to antibiotic decision-making, including an individualistic and hierarchical culture, may limit integrated care in surgical pathways^14^. Efforts are now required at all scales to establish roles, responsibilities and accountabilities of surgical and anaesthesia providers in infection prevention and control (IPC), diagnosis of infections and antibiotic prescription, for a sustainable improvement of antibiotic use. Optimizing current practice and implementing appropriate AMS initiatives through collaborative multidisciplinary teams is likely to remain fundamental to minimize SSI and mitigate AMR. Approaches that include patient and carer participation and cooperation, nurse leadership, empowering pharmacists, engaging surgical and anaesthetic leads is essential.

Technological and medical developments have had a profound effect on surgery over the last few decades. The change to primarily non-operative management of many conditions (for example, appendicitis or diverticulitis) are resulting in the increasing use of antimicrobials. The developments in minimally invasive procedures, robotics, artificial intelligence and digital technologies promise a future where surgery is more personalized, less invasive with faster recovery times, shorter hospital stays and lower risk of harm. Preoperative and follow-up assessment will therefore acquire even greater importance. Recognizing that much of surgical care is delivered in environments without experts in infectious diseases, antimicrobial stewardship, microbiology and infection prevention, virtual platforms may be leveraged to assist rural environments and low-resource settings through real-time expert consultation *via* telemedicine and improved decision support systems. With increasing day-care surgery and shorter hospital stays, digital platforms with patient’s generated data are used for the postoperative follow-up. In the era of the electronic medical records, machine learning can efficiently predict the risk of postoperative infection and resistance, which can then assist prioritizing interventions for patients at greatest risk, guide on treatment options, and minimize unnecessary exposure to antibiotics. Advances in decision support and artificial intelligence in postoperative infection diagnosis and management have particular relevance for LMICs. Personalized and more targeted antibiotic prescribing will also minimize the disruption of the human microbiome to maximize its protective effect against resistant bacteria and antibiotic-associated diarrhoea (for example, *Clostridium difficile* infections).

To ensure safe surgical care, addressing AMR must be integral in any surgical planning or surgical strategy and must be considered a vital component of service resilience. The use of new technologies must include a greater emphasis on implementation, and on behavioural and organizational science to better influence reliability in application and adoption. Broad, collaborative, multidisciplinary efforts with creative thinking are fundamental to mitigate AMR and limit postoperative infections with resistant organisms.

## Disclosure

The authors declare no conflict of interest.
